# Endogenous Cushing’s Syndrome Due to Right Adrenal Adenoma Presenting With Bilateral Leg Oedema and Skin Ulceration

**DOI:** 10.7759/cureus.71103

**Published:** 2024-10-08

**Authors:** Morika Suzuki, Takashi Watari

**Affiliations:** 1 Department of General Internal Medicine, National Hospital Organization Sendai Medical Center, Miyagi, JPN; 2 General Medicine Center, Shimane University Hospital, Izumo, JPN

**Keywords:** adrenal adenoma, cushing’s syndrome, erythema, leg edema, skin ulceration

## Abstract

Cushing’s syndrome (CS) is a rare disorder characterized by an excess of glucocorticoids, leading to distinctive clinical manifestations. However, its presentation can be atypical, complicating diagnosis. We describe a 53-year-old woman's case of endogenous, adrenocorticotropic hormone (ACTH)-independent CS, presenting with bilateral leg edema and shin ulceration, without the classic signs of moon face or truncal obesity initially. Despite being treated for hypertension and dyslipidemia for a decade, and having a history of left capsular hemorrhage, the diagnosis of CS was not considered until extensive testing was conducted. Investigations revealed a low ACTH level, elevated urinary free cortisol, and a right adrenal mass, leading to the diagnosis of CS caused by an adrenal adenoma. Following laparoscopic adrenalectomy, the patient's symptoms rapidly improved. This case underscores the importance of considering CS in the differential diagnosis of edema and skin ulceration, especially when clinical presentations deviate from the norm. It highlights the complexity of CS pathogenesis and the need for thorough evaluation to prevent misdiagnosis, emphasizing that not all patients will present with or recognize the classic.

## Introduction

Cushing’s syndrome (CS) is a rare systemic disease caused by a chronic excess of glucocorticoid [1]. The most common cause is exogenous glucocorticoid administration [1]. Endogenous CS is classified as adrenocorticotropic hormone (ACTH) dependent (80%) and ACTH independent (20%) [1-3]. The characteristic clinical findings of CS, such as moon face, purple abdominal striae, and proximal muscle atrophy in the limbs, are not always present, so the diagnosis can be missed [1-3]. We report a case of endogenous CS that presented as bilateral leg edema and skin ulceration.

## Case presentation

A woman in her 50s presented with bilateral leg edema that had been present for three years and a skin ulcer on her shin that had been present for two months. She had been on treatment for hypertension and dyslipidemia for 10 years and had been hospitalized for a left capsular hemorrhage three years back. She was not receiving any exogenous steroids, including topical formulations.

**Figure 1:**
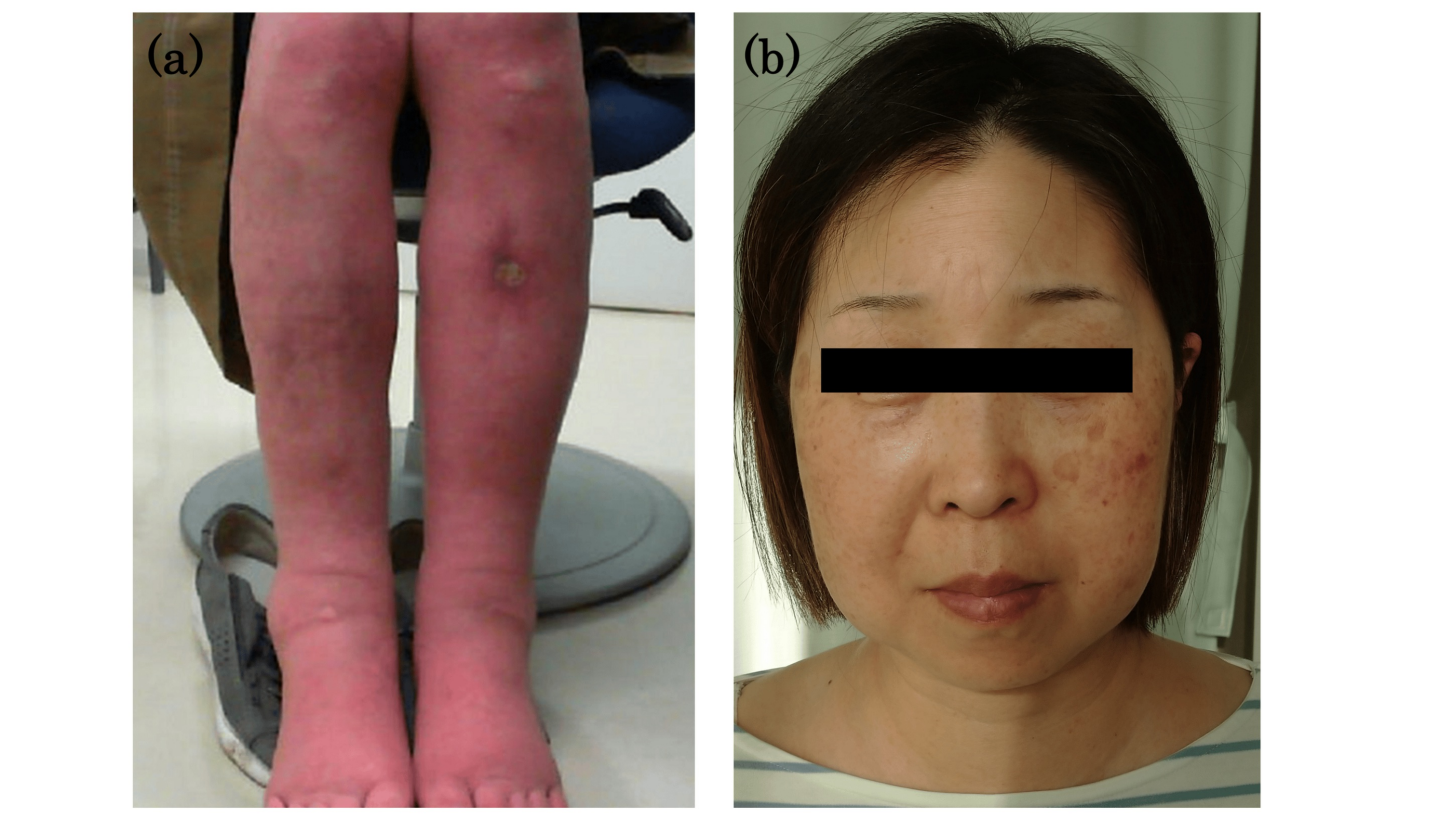
The patient’s lower legs and her face. (a) The patient’s lower legs showing symmetrical pitting edema, an erythematous purpuric rash, and a 1.3 cm round ulcer on the left shin. (b) The patient's face has a characteristic “moon” appearance with round cheeks and some redness. In addition, bilateral fat accumulation is present in the temporal and supraclavicular areas.

The patient’s height, weight, body mass index (BMI), waist circumference, and waist-to-height ratio were 160 cm, 60 kg, 23.4 kg/m2, 88.5 cm, and 0.55, respectively. Her vital signs were stable. Both lower legs had pitting edema with erythema and purpura, and she had a 1.3 cm circular ulcer on the left shin (Figure 1a). The physical examination findings of moon face (Figure 1b), truncal obesity, and thinning of the skin on the lower legs led us to consider CS in the differential diagnosis.

**Figure 2:**
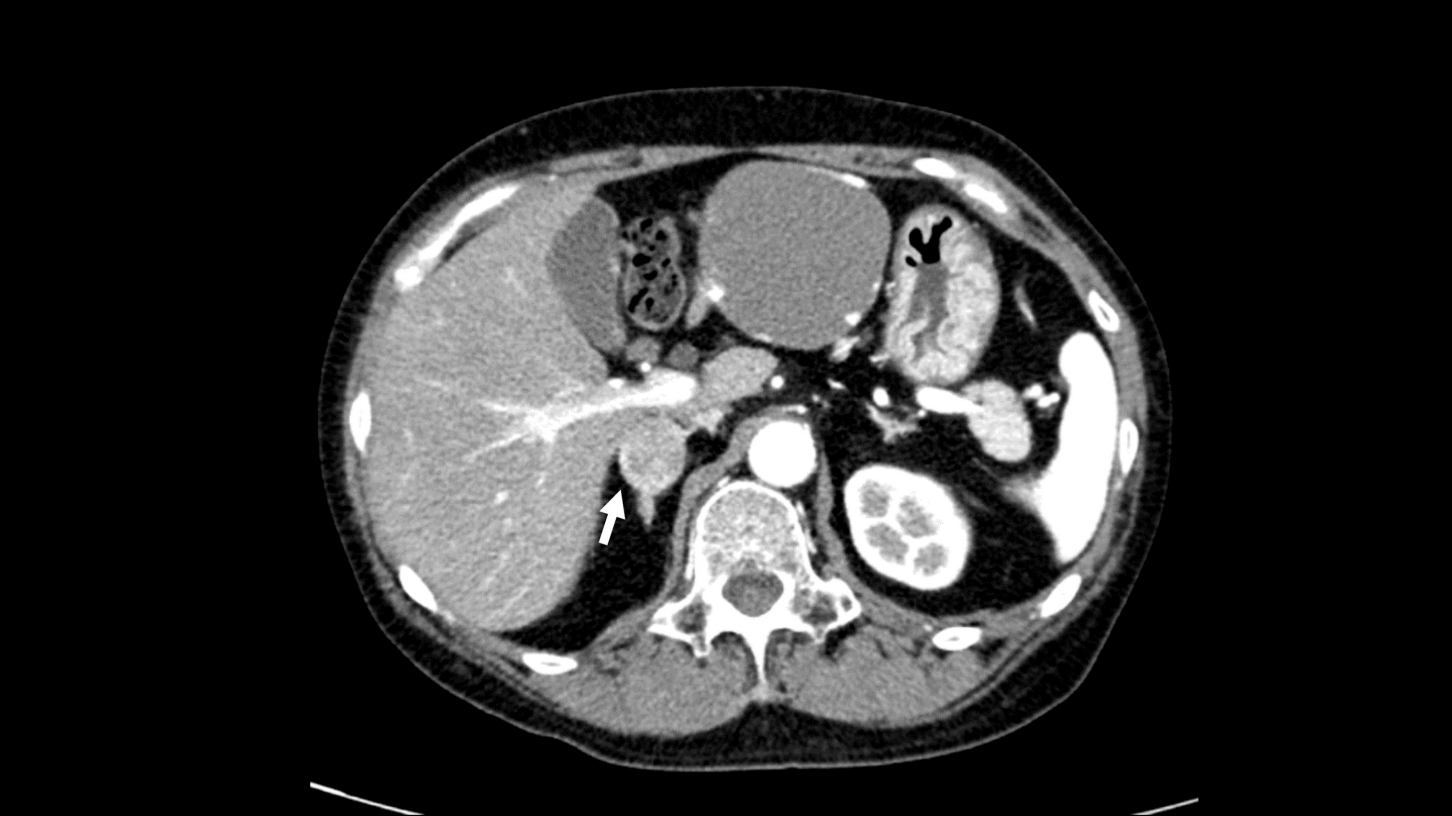
Contrast-enhanced computed tomography imaging showing a 23 mm low-absorption mass in the right adrenal gland.

Her blood and urine test results were normal. Computed tomography revealed a 23 mm low-absorption mass in the right adrenal gland (Figure 2). The CT scans identified a mass with Hounsfield units averaging 20±15 HU. A contrast medium was used during these scans to enhance visibility.

The physical examination findings of moon face, truncal obesity, and thinning of the skin on the lower legs led us to consider CS in the differential diagnosis. It is important to clarify that although an incidental adrenal tumor protocol was not strictly followed, the imaging studies were tailored toward a Cushing’s syndrome (CS) diagnosis. This was due to clinical findings suggestive of CS, which had been initially overlooked. As a result, the CT scans followed the diagnostic algorithm for CS rather than that for a typical incidental adrenal tumor.

Endocrine test results revealed a low blood ACTH level (<1.5 pg/mL; normal: 7.2-63.3 pg/mL), normal blood cortisol level (17.0 μg/dL; normal: 2.9-19.4 μg/dL), and an elevated urinary free cortisol level (214.2 μg/day; normal: 11.2-80.3 μg/day). A 1 mg dexamethasone suppression study did not suppress the next morning's blood cortisol level (14.8 μg/dL; normal: ≤5 μg/dL), and late-night blood cortisol was elevated (16.2 μg/dL; normal: ≤7.5 μg/dL). ¹³¹I-aldosterol scintigraphy showed hyperaccumulation in the right adrenal gland and suppression in the left adrenal gland. Based on these findings, we diagnosed ACTH-independent endogenous CS due to a right adrenal tumor. Laparoscopic right adrenalectomy was performed. Histopathology confirmed the diagnosis of adrenal adenoma. Postoperatively, the leg edema and erythema rapidly improved, and the purpura and ulceration improved after one month. One year after the surgery, blood tests showed a cortisol level of 5.4 μg/dL and an ACTH level of 73.1 pg/mL (measurement was performed in the morning outpatient clinic), and her blood pressure was well controlled. Thus, her antihypertensive medication was discontinued, and she was instructed to visit her family doctor regularly.

## Discussion

In this case, we did not initially consider CS in the differential diagnosis because the patient’s presenting complaints were chronic leg edema and leg ulceration. She did not report changes in her facial appearance or body shape, which made it easy to overlook her moon face and truncal obesity as signs of CS. The diagnostic efficiency and timeliness were compromised because she did not present with the typical textbook symptoms and signs of CS, and because the patient did not perceive her abnormal facial appearance or body shape as abnormal, representing a classic pitfall in diagnosis. The clinical presentation of CS is varied, and the frequency of each clinical manifestation is not established. However, reports suggest that moon face and truncal obesity are observed in approximately 90% of cases [4,5]. However, weight gain, excessive abdominal fat, and general fatigue are common in the general population and are not specific. Non-specific clinical manifestations such as hypertension, abnormal lipid and glucose metabolism, edema, and increased susceptibility to infections due to excessive glucocorticoid levels make the disease difficult to recognize [1,4].

Additionally, the pathogenesis of edema in CS is complex. It is thought to be caused by sodium retention and increased vascular permeability due to excess glucocorticoid levels. The edema can be confined to the lower legs, as in this case, or can be generalized. Edema is reported in 15% of patients with CS [5]. Patients with CS often have hypertension and obesity, contributing to the complexity and variability of edema, making quantification based on patient reports difficult. Edema may be present in more cases than reported. Although CS may not come to mind when confronted with a case of edema, it should be considered when the cause of edema is unclear.

Skin thinning in CS is caused by glucocorticoid-induced inhibition of epidermal cell division and dermal collagen synthesis [6,7]. This leads to increased vascular fragility, resulting in subcutaneous hemorrhage, purpura, and delayed wound healing [6,7]. In this case, blood flow stasis due to edema further impaired wound healing, leading to ulceration. Furthermore, weight gain and abdominal fat deposition are common in CS, but findings due to the increasing prevalence of metabolic syndrome related to global obesity resemble the clinical picture of CS due to glucocorticoid excess, complicating the diagnosis [1,4,8]. Distinguishing CS from simple obesity requires the presence of signs such as osteopenia, thin skin, and ecchymoses due to glucocorticoid excess [1,4,5]. The frequency of non-obese patients with CS is unknown. In this case, the presence of abdominal fat deposition without obesity made it even more difficult to recognize the disease. In summary, clinicians should be aware that patients with CS may present with chronic leg edema and skin abnormalities. Especially when wound healing delay or ulceration is observed as part of the clinical picture, the possibility of skin thinning due to CS should be considered, and CS should be included in the differential diagnosis. However, it is difficult for physicians to notice chronic changes in facial appearance and body shape if the patient does not report them. Recognizing that patients themselves may not perceive these changes as pathological, recalling CS as a differential diagnosis based on other findings and history, conducting thorough interviews and examinations, and not missing characteristic findings are crucial for diagnostic excellence.

## Conclusions

It is easy to overlook important clues to the diagnosis when the clinical presentation does not align with the classic textbook description of CS and when patients do not consider characteristic signs such as moon face and trunk obesity as abnormal, as in this case. It is important to consider CS in the differential diagnosis of edema and skin ulceration. Physicians should be aware that patients may not recognize changes in facial appearance and body shape as pathological. An in-depth interview and examination should be conducted to avoid missing any characteristic findings.
